# Notes and News

**Published:** 1896-04-18

**Authors:** 


### Notes and News.

(Collected and Communicated.)

### HOSPITAL MEETINGS.

A meeting of Governors of the Hospital for Epilepsy and Paralysis, Regent's Park, will be held on the 21st instant at 3 p.m.

The Annual Festival Banquet of the Royal Eye Hospital, Southwark, will take place at the Whitehall Rooms, Hotel Metropole, on Tuesday, April 21st, at 7 p.m., Mr. James Bailey, M.P., in the chair.

The Biennial Dinner in aid of the Hampstead Hospital, Parliament Hill Road, N.W., will be held on Thursday, May 7th, at the Grand Hotel, Northumberland Avenue, W. F. Malcolm, Esq., in the chair.

The new hospital for Massachusetts is to be erected in Rutland, a town in the centre of the State standing 1,200 feet above the level of the sea.

In view of the large and increasing number of deaths resulting from the use of dangerous lamps, the Home Secretary proposes to secure the re-election of the Select Committee appointed in 1894 to inquire into the matter.

It may be admitted that medical journalism is privileged in its phraseology, but overstepping the bounds of good taste should nevertheless be avoided. A description of the opening of an operation theatre, which appeared in an American contemporary, contains the following: "The first blood in the operating theatre was from," &c. This is a style of descriptive journalism which is quite inexcusable.

A most useful institution in France appears likely to fall into disuse owing to a system of overstrict regulation. This institution is a convalescent home given to the French Government for the use of officers by Madame Furtado Heine, and endowed by her with an income of £2,400 yearly. It was intended to provide ease and comfort at a small expense for officers recovering from illness; but as a sort of military discipline is maintained, the first invalids to arrive found the conditions so uncomfortable that they left it.

A new iron building for the reception of casualties is to be opened, in connection with the Miller Hospital, on Saturday, by Mr. Passmore Edwards.

The Earl of Dartmouth has given £25 towards necessary alterations in the operating theatre of the Wolverhampton and Staffordshire General Hospital.

A convalescent home, in connection with St. Giles Christian Mission, has just been opened at Hastings. The building is also to be used as a holiday home for children.

It is stated that a hospital has been destroyed in Cuba by the Spanish troops. The patients escaped, but the portable contents of the hospital were seized by the troops before the destruction of the building.

Mr. Peter Coats, of Ferguslie Thread Works, has promised the magnificent donation of £10,000 towards a new hospital for Paisley. This sum will cause his donations in connection with the hospital to reach £25,000.

The Jacksonian Prize has been awarded by the Council of the Royal College of Surgeons to Dr. A. A. Kanthack for his essay on "Tetanus." At the same meeting the Walker Prize for the best work done within the last five years in advancing the knowledge of the pathology and therapeutics of cancer was awarded to Mr. Harold Jelland Stiles, M.B.C.M. Edin.

During the past year collections have been made at Sheffield to secure a memorial of Dr. Cleaver, one of the founders of the Sheffield Children's Hospital, where he acted as treasurer and senior medical officer. Chiefly through the exertions of the ladies' committee of the hospital, the fund now amounts to £668. It has been decided to devote this sum to the erection of a new ward in connection with the institution, for which purpose further subscriptions will be necessary.

We have never heard that dentistry and medicine were antagonistic to each other in England. In America, however, apparently they are not always on the best of terms. Quite recently, at Philadelphia, the students of the Dental College and those of the Medico-Chirurgical College had a pitched battle. The encounter took place in one of the halls, and the battle only ceased on the arrival of the police, and not before several of the combatants were seriously injured.

In a recent number of *Truth*, our frequent warning against collections of old postage stamps for charitable purposes is endorsed. The fallacy of securing anything but the most ridiculous price from a dealer is insisted upon. No charity is the better for such absurd practices, and the trouble which friends are put to could be easily prevented by asking them for sixpence or a shilling outright, which sum would be gladly given, we should imagine, in place of the thousands of stamps such a sum would fetch. These collections are growing to be a mania with people who have few interests; and we have heard of one lady who is colle ting for an unnamed charity, but, failing to find the institution, is content to think that her collection may secure her a new pair of gloves.

